# Les complications tardives de prothèse totale de la hanche: à propos de 42 cas

**DOI:** 10.11604/pamj.2013.14.17.2265

**Published:** 2013-01-12

**Authors:** Mohamed Azarkane, Hassan Boussakri, Mohamed Shimi, Abdlehalim Elibrahimi, Abdlemeji Elmrini

**Affiliations:** 1 Service de chirurgie orthopédique et traumatologie B4, CHU Hassan II, Fes, Maroc

**Keywords:** Prothèse totale de la hanche, complications tardives, total hip prosthesis, late complications

## Abstract

L'arthroplastie de la hanche est un moyen fiable dans le traitement des affections de la hanche. En lui rendant sa mobilité sa stabilité et son indolence. Cependant cette chirurgie prothétique expose au risque de la survenue des complications qui peuvent engager le pronostic fonctionnel. Nous avons réalisé une étude rétrospective sur une durée de 8 ans de janvier 2004 au janvier 2012 au service de traumatologie-orthopédie de CHU HASSAN II FEZ. Pendant cette période nous avons opéré 240 patients pour PTH. Après un recul moyen de 5 ans nous avons noté chez 42 (17,4%) patients une complication tardive. Nous noté 13 cas de descellement aseptique soit 5,4%. Cette complication a été survenue dans notre série sur une prothèse cimentée dans 8 cas et non cimentée dans 5 cas. Le traitement que nous avons adopté dans notre série a été une reprise de PTH sans greffe osseuse ni anneau de reconstruction dans 4 cas, reprise avec mise en place d'anneau de Kerboull dans 7 cas et reprise avec greffe osseuse et anneau de kerboull dans 2 cas. Nous avons trouvé 11 cas de sepsis tardive soit 4,6% des cas. Nous avons le diabète comme facteur de risque chez 3 malades. L'agent causal a été staphylococcus épdermidis dans 5 cas, colibacille dans 2 cas et association staphylococcus-BGN dans 1 cas. Les différentes modalités que nous avons utilisé pour traiter l'infection dans notre ont été un lavage simple, système d'irrigation-drainage et réimplantation simple en un seul temps ou en 2 temps avec couverture systématique par une antibiothérapie adaptée selon l'antibiogramme. Nous avons noté également 11 cas de fracture sur PTH intéressant dans tous les cas le fémur, nous avons traité ce type de fracture dans notre série par une tige fémorale prothétique longue dans 4 cas, une plaque vissée cerclée dans 3 cas et cerclage simple dans 4 cas. La consolidation a été obtenue chez 9 patients avec 2 cas de pseudarthrose. Nous avons noté 7 cas de luxation tardive de PTH. Comme facteur de risque dans notre série nous avons trouvé le sexe féminin et le surpoids. Sur le plan technique la malposition de cotyle a été constituée l'étiologie principale avec 4 cas. Nous avons traité les cas de luxation par réduction simple avec traction dans 3 cas et une reprise chirurgicale pour corriger la malposition de cotyle dans 4 cas. Nos résultats sont comparables avec ceux de la littérature. Selon les résultats de la littérature le descellement aseptique constitue la complication la plus fréquente. Pour traiter cette complication les 2 modalités la plus fréquemment utilisées dans a littérature sont la reprise avec des greffes et anneaux de reconstruction ou fixer la nouvelle cupule sur os sain de néocotyle créé par descellement. Le résultat de la littérature objective aussi la responsabilité de staphylococcus comme agent causal la plus fréquent. Il montre également l'efficacité de traitement chirurgical par réimplantation de la prothèse en un seul ou en 2 temps. L'étude de la littérature objective aussi que La prise en charge des fractures sur PTH est difficile en raison de l'âge souvent avancé et de la fragilité des patients, de l'ostéoporose, et de la menace que ces fractures font peser sur la fixation de la prothèse parfois déjà défaillante. Les complications tardives de PTH sont fréquentes et sont causes de reprises de chirurgie prothétique et rendent leur prise en charge très difficile. Elles peuvent transformer les légitimes espoirs fonctionnels en catastrophe invalidante.

## Introduction

Les complications tardives de prothèse totale de la hanche (PTH) sont fréquentes et sont causes de reprises de chirurgie prothétique et rendent leur prise en charge très difficile. Elles peuvent transformer les légitimes espoirs fonctionnels en catastrophe invalidante laissant des séquelles parfois plus importante que la gêne qui l'avait motivée. Grace à cette étude nous allons essayer de détecter les différentes complications tardives qui peuvent survenir chez un patient qui porte une prothèse totale de la hanche.

## Patients et observations

Nous avons mené une étude rétrospective de durée de 8 ans de janvier 2004 au janvier 2012. Nous avons étudié 240 dossiers des malades opérés pour PTH. Et nous avons noté une complication tardive chez 42 cas (17,5%). Les objectifs de notre étude étaient de détecter les complications à long terme de chirurgie prothétique de la hanche; de chercher un éventuel facteur de risque; d'identifier le traitement qu'on peut utiliser pour prendre en charge de ces types des complications.

**Critères d'inclusion**: Les malades opérés pour PTH soit en première intervention ou reprise et qui ont présenté une des complications tardives quelque soit la cause qui a motivé l'intervention pour la mise en place de la prothèse.

**Critères d'exclusion**: Les malades perdus de vu, non suivis ou non traités après le diagnostic de la complication. Les malades traités pour PTH et qui avaient une bonne évolution clinique et radiologique.

**Epidémiologie**: L'âge moyen dans notre série était 45 ans, l'atteinte de sexe masculin était prédominante 65%. L'indication de la prothèse initialement est incluse dans le Tableau 1.

**Tableau 1:** Les indications de prothèse totale de la hanche dans notre

Indication initiale	Nombre de cas	%
Coxarthrose	15	35,7%
Coxite inflammatoire	11	26,2%
Fracture de col fémoral	7	16,6%
Nécrose aseptique	4	9,5%
Dysplasie de cotyle Coxalgie	3	7,1%

**Technique chirurgicale**: La voie d'abord utilisée dans série était de type: postéro-externe de Moore dans 18 cas; postérieure mini-invasive dans 24 cas. tLe type de prothèse utilisé avait un couple frottement méthal-plythéline, cimentée dans 22 cas, non cimentée dans 15 cas et hybride dans 5 cas

**Recul**: Le suivi des patients dans notre série était clinique et radiologique, avec un recul moyen de 5 ans. Dans une série de 240 patients traité pour PTH, nous avons trouvé chez 42 cas une complication tardive (Tableau 2)

**Tableau 2:** Le type de complications et leur fréquence dans notre série

Complication	Nombre de cas	%
Descellement aseptique	13	5,4%
Sepsis tardif	11	4,6%
Fracture sur prothèse totale de hanche	11	4,6%
Luxation tardive	7	3%

### Complications

**Figure 1:**
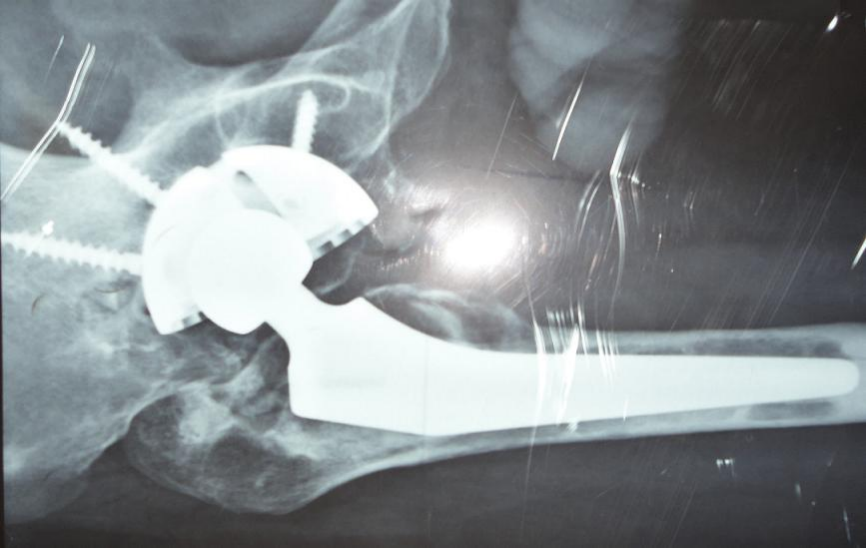
Radiographie standard d'un patient qui présente un descellement aseptique bipolaire

**Figure 2:**
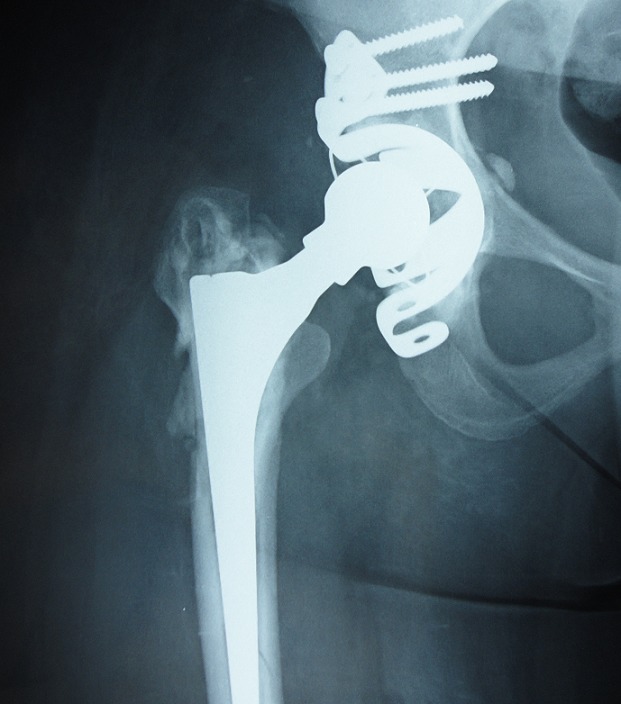
Radiographie de contrôle après reprise avec recèlement et reconstruction du cotyle par la mise en place de la croix de Kerboull

**Descellement aseptique (Figure 1, Figure 2)**: est une complication fréquente et grave, nous avons noté 13 cas de descellement aseptique dans notre série soit 5,4%. Il intéressait le cotyle dans 11 cas et la tige fémorale dans 2 cas, il était survenu sur une prothèse cimentée dans 8 cas et non cimentée dans 5 cas. Il était classé selon la classification de VIVES et COLL pour le cotyle: stade I: 4 cas, stade II: 5cas et stade III: 2 cas. Et selon la classification de Delee et Charnley il était siège au niveau de la zone I: 8 cas, Zone II: 5cas et zone III: 2cas. Le traitement adopté dans notre série était une reprise sans greffe et sans anneau associé dans 4 cas, reprise sans greffe avec anneau de reconstruction de type de Kerboull 7cas et reprise avec greffe osseuse et anneau de Kerboull dans 2 cas. Pour le descellement de fémur nous avons réalisé un recèlement simple dans les 2 cas.

**Figure 3:**
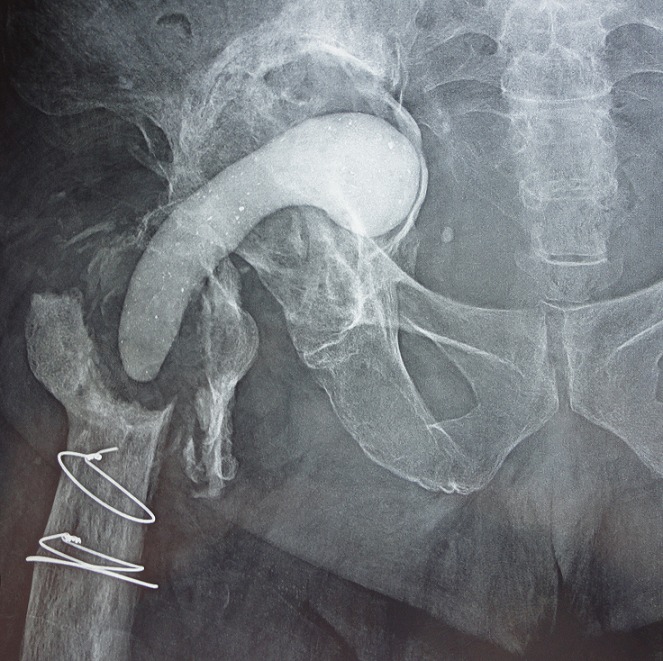
Radiographie stand d'un patient qui présente un sepsis tardif sur prothèse totale de la hanche avec ablation de prothèse et mise en place d'un spacer au ciment antibiotique

**Figure 4:**
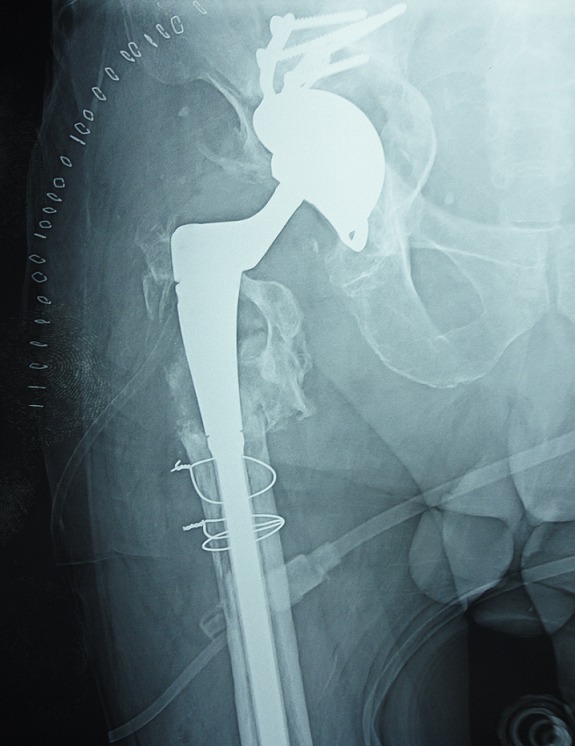
Radiographie de contrôle après reprise après 2 mois

**Infection (Figure 3, Figure 4)**: La survenue d'une infection sur une PTH est une complication très grave, mais également difficile a gérer, dans notre série nous avons noté 11 cas de sepsis tardif (4,6%), les implants de prothèse infectée sont cimentés dans 7 cas pour la cupule et non cimentés dans 4 cas. Comme facteur de risque nous avons noté le diabète chez 3 malades. Cliniquement tous les patients ont présenté une douleur au niveau de la hanche opérée, la fistule productive dans 5 cas et les signes inflammatoires locorégionaux dans 2 cas. Sur le plan radiologique: nous avons noté 3 cas de descellement de cotyle et 1 cas de descellement bipolaire, et des géodes dans 2 cas. La CRP était positive chez 7 patients. L'étude bactériologique a été réalisée chez 3 patients, qui ont une fistule productive, et son résultat était négatif chez 2 patients, mais elle a mis en évidence le *Staphylococcus epidermidis* chez un patient. Le traitement que nous avons adopté dans notre série a été chirurgical chez tous les patients avec un prélèvement bactériologique systématiquement dans tous les cas: nettoyage simple: 3 cas; irrigation-lavage: 2 cas; ablation de cotyle: 6 cas; réimplantation de la prothèse en un seul temps: 3 cas; réimplantation de la prothèse en 2 temps avec intervalle libre de 6 semaines chez 2 patients et 2 mois pour le 3e patient.

Le prélèvement bactériologique en peropratoire était systématique chez tous les malades, son résultat a objectivé *Staphylococcus epidemidis* dans 4 cas; et colibacille dans 2 cas et association staphyloccocus et BGN dans 1 cas. Nous avons complété par une antibiothérapie adaptée au résultat de l'antibiogramme pendant au moins 6 semaines en général à base de fluoroquinilones.

Nous avons évalué la guérison dans notre série selon des critères cliniques, biologiques et radiologiques après un recul de 2 ans nous avons noté: la récidive de l'infection chez 2 patients traités initialement par des nettoyages simples à répétition.

### Fracture sur PTH (Figure 5, Figure 6)

Dans notre série nous avons noté 11cas de fracture de fémur sur PTH, la fracture était survenue sur une prothèse non cimenté dans 7 cas et prothèse cimenté dans 4 cas. L'atteinte de sexe féminin dans 7 cas. Nous avons noté comme facteur de risque l'ostéoporose chez 4 patients et sexe féminin. Nous avons utilisé la classification de Vancouver qui repose sur la localisation de la fracture et selon cette classification nos avons trouvé: fracture de type B2 dans 4 cas et de type C dans 7cas. Le traitement adopté dans notre série était une mise place d'une tige longue dans 4 cas, plaque vissée avec cerclage dans 3cas et cerclage simple dans 4 cas.

**Figure 5:**
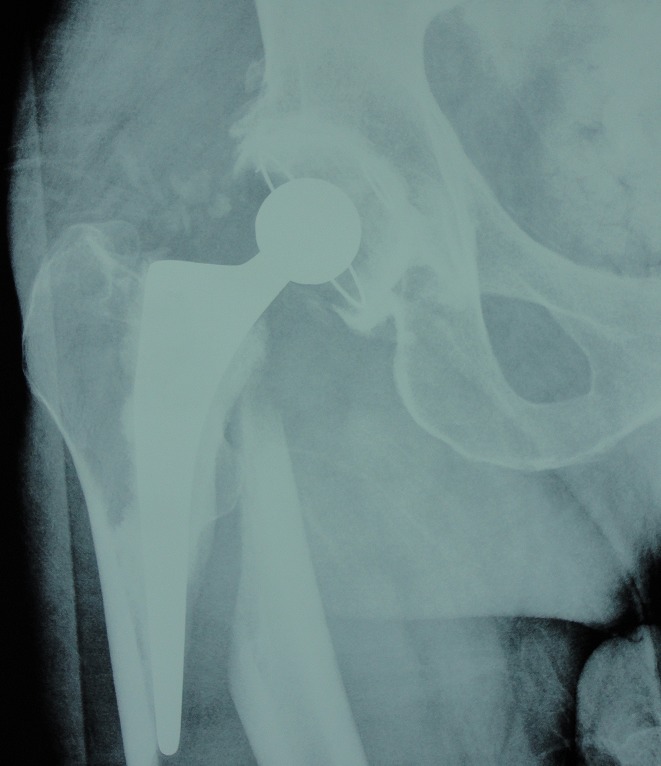
Radiographie de la hanche d'un patient qui présente une fracture sur prothèse totale de la hanche classée stade B selon la classification de Vancouver

**Figure 6:**
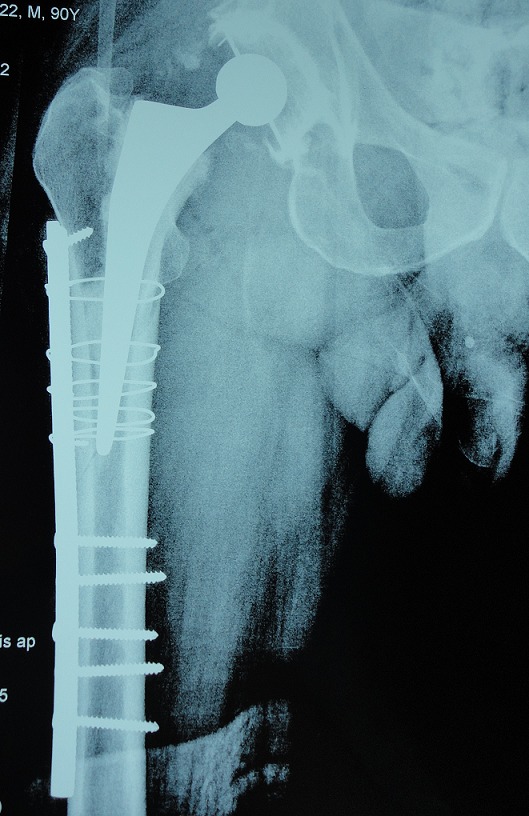
Radiographie de contrôle après ostéosynthèse par une plaque vissée et cerclée

### Luxation tardive sur prothèse (Figure 7)

C'est la survenue de la luxation sur une prothèse totale de la hanche au-delà de 5 ans. C'est une complication fréquente. Dans notre série de 240 prothèses nous avons trouvé 7 cas de luxation tardive (3%). L'atteinte de sexe féminin est prédominante 5 femme et 2 hommes. L'indication de PTH était initialement: gonarthrose dans 4cas, 2 cas de coxite inflammatoire et un cas de dysplasie de cotyle. Le cotyle était cimenté dans 4 cas et non cimenté dans 3 cas. Les facteurs de risque que nous avons détecté dans notre série étaient surtout le sexe féminin (5femmes/ 2 hommes) et le surpoids. Sur le plan technique la malposition de cotyle constitue la cause principale 4 cas, et aucun cas de malposition de la tige fémorale. Sur le plan thérapeutique: la réduction était réalisée sous AG dans 3 cas compléter par une traction pendant 6 semaines puis la rééducation. Nous avons noté la récidive de luxation dans 1 cas. Le traitement était chirurgical dans 4 cas: changement de position de cotyle dans 3 cas: dans 2 cas le cotyle était cimenté dont son ablation était difficile mais sans dégâts osseux. Nous avons réalisé le changement de position et de l'orientation de cotyle dans les 3 cas avec réimplantation au niveau de son ancienne cavité sans anneau de reconstruction ni greffe osseuse. Nous avons noté une bonne évolution des patients traités. Nous avons réalisé dans un cas une reconstruction de cotyle par anneau de Kerboull chez un patient traité par PTH pour dysplasie du cotyle.

**Figure 7:**
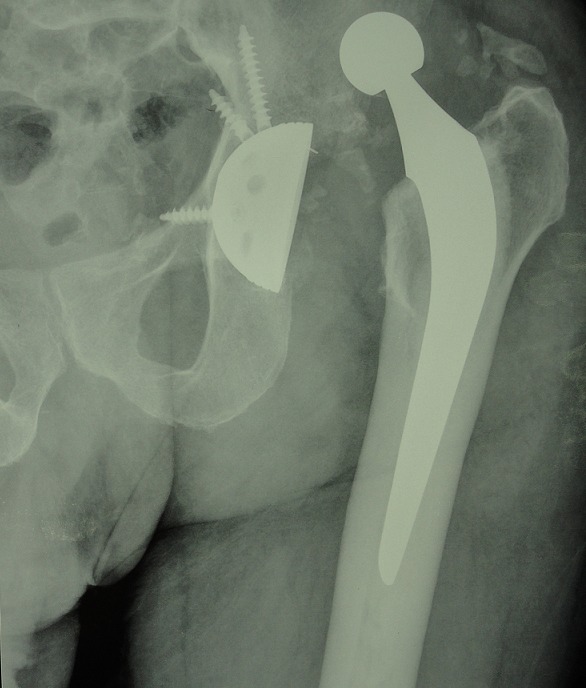
Radiographie standard de la hanche montre une luxation de prothèse totale de la hanche, la malposition de cotyle constitue la cause principale

## Discussion

### Descellement aseptique

Il constitue le problème évolutif le plus préoccupant des arthroplasties totales de la hanche, cette faillite conduit tôt ou tard à une révision rendue difficile par la dégradation de tissu osseux de soutien. Dans notre série nous avons trouvé 13 cas de descellement de cotyle dans 240 cas soit 5,4% et 2 cas de descellement de la tige fémorale (Tableau 3) Les études de la littérature ont objectivé que le descellement de cotyle est responsable de 13% de reprise de PTH [4]. Pour reconstruire on a 2 possibilités: certaines équipes [5] préconisent des greffes avec anneaux de reconstruction; d'autre adoptent: fixer la nouvelle cupule sur os sain de néocotyle créé par descellement ce qui ne permet pas toujours de descendre suffisamment le centre de rotation [6] Dans notre série nous avons traité 13 cas de descellement aseptique: fixation de la nouvelle cupule sur l'os sain au niveau de néocotyle créé par le descellement: 4 cas; utilisation d'anneau de kerboull sans greffe osseuse: 7 cas; anneau de kerboull avec greffe de l'os spongieux: 2 cas. Nous avons noté 3 cas de reprise: 2 cas traités par la fixation de nouvelle cupule sans anneau de reconstruction ni greffe osseuse en rapport avec un descellement itératif après un recul de 3 ans, un cas traité par reconstruction de cotyle par anneau de kerboull due à une mauvaise fixation de croix de kerboull. Les résultats du scellement itératif cotyloïdien sans greffe osseuse ni anneau de soutien rapportés dans la littérature sont peu favorable puisqu'ils oscillent entre 10% et 51% de descellements itératifs selon les séries et le recul (Tableau 4).

**Tableau 3:** La fréquence de descellement dans les différentes séries

Série	Nombre de prothèse totale de hanches	Recul moyen (années)	% descellement
Lee BP [1]	126	10	5%
Ray A [2]	1000	10	37%
Ulf Riede [3]	161	15	10,55%
Notre série	240	5	5,4%

**Tableau 4:** La fréquence descellement selon les séries

Auteur	Recul	% de descellement itératif
Amurtz HC [7]	2 ans	10
Hunter GA [8]	6 mois	51
Raut VV [9]	7 ans	36,6
Russotti GM, [10]	11 ans	16
Notre série	3 ans	2 cas

### Infection

L'infection est une complication grave et fréquente et sa prise en charge est très difficile (Tableau 5). On parle de l'infection lorsqu'un au moins des critères suivant est présent [13]:

- Présence de pus
- Germe isolé au niveau de site
- Signes évidents d'infection au niveau de site
- Diagnostic clinique posé par le chirurgien
- Présence de pus
- Germe isolé au niveau de site
- Signes évidents d'infection au niveau de site
- Diagnostic clinique posé par le chirurgien

**Tableau 5:** La fréquence de l'infection dans les différentes séries

Série	Fréquence
Connault P [11]	1,2%
Solomon DH [12]	0,4%
Notre série	4,6%

La responsabilité de l'infection incombe essentiellement aux cocci gram + surtout Staphylococcus epidermidis, résistant ou sensible à la méthicilline. Les bacilles gram négatifs sont faiblement représentés avec prédominance de colibacille. On peut trouver également les corynébacteries, les bacteroides, les clostrodiums et les myobateries. Alors que dans notre étude nous avons détecté comme germe responsable *Staphyloccus épidermidis* dans 5 cas et colibacille dans 2 cas et association staphyloccocus et BGN dans 1 cas.

Les études de la littérature ont trouvé que dans 81% des cas l'infection a été considérée monobactérienne ce chiffre est proche à ce que nous avons trouvé dans notre étude 85%. Dans 15% des cas deux germes ont été considérés comme responsables [14].

Pour détecter le germe responsable l'étude bactériologique est primordiale. Ainsi ce sont les prélèvements préo-opératoires profonds qui sont les plus fiables lorsque les prélèvements multiples sont réalisés, et les frottis superficiels et les ponctions des parties molles devraient sans doute abandonnés [14].

La prise en charge d'une PTH infectée est chirurgicale [13], le traitement médical est aussi fondamental que l'excision chirurgicale, les principes de traitement médicale [15]:

- La bithérapie est plus efficace que la monothérapie
- Un temps de traitement prolongé est indispensable pour obtenir la stérilisation de foyer d'infection
- Les molécules choisies doivent être en fonction de l'antibiogramme mais aussi il faut intégrer aussi le tropisme osseux de l'antibiotique
- Les doses doivent être fortes
- Le mode d'administration dépend de tropisme osseux
- La durée de l'antibiothérapie doit être longue au moins un mois

### Le traitement chirurgical de l'infection de la PTH

**Excision lavage et antibiothérapie avec conservation de la prothèse**: la revue de la littérature [16] a montré que les résultats de cette méthode sont mauvais. Cette technique n'est plus adaptée, elle est utilisée surtout dans les infections aigues ceci était confirmé par notre étude où nettoyage seul associé aux antibiotique était utilisé dans 3 cas et n'avait donné une guérison totale au recul qu'à un seul cas.

**Réimplantation de la prothèse en un seul temps**: ablation de la prothèse et nettoyage et repose de la prothèse cette technique est plus adoptée par plusieurs [17] auteurs qui évite au patient une 2éme intervention et l'inconfort de la période entre les 2 temps, nous avons utilisé cette technique dans 3 cas et nous avons obtenu la guérison dans tous les cas. Les résultats de la littérature a montré que le taux de guérison est 88% mais il n'était pas influencé par la présence de pus franc, de la fistule, l'état clinique préopératoire, le stade de descellement, le germe responsable et le type d'implant de révision.

**Réimplantation en 2 temps avec intervalle libre sans prothèse**: Cette technique est adopté par plusieurs auteurs [18, 19], elle consiste à une ablation de prothèse avec nettoyage et exérèse de tissu nécrotique et mise en place d'un espaceur souvent en ciment antibiotique, puis la mise en place de la prothèse après un intervalle libre variable en fonction de l'évolution de l'infection. Dans notre série nous avons adopté cette technique dans 3 cas. On n'a pas noté des lésions osseuses après l'ablation de la prothèse contrairement a à ce que nous avons trouvé dans la littérature [20] après l'analyse des stades de SOFCOT peropétoires met en lumière une nette aggravation des lésions osseuses lors de l'ablation des implants puisque 30 acétabulums et 20 fémurs passant de stade I aux stades III et IV. Le résultat de cette technique est satisfaisant avec guérison quasi complète dans tous les cas traités dans notre série. Alors que le résultat de la littérature avait montré que le taux de guérison est 85% légèrement inférieur à celui de réimplantation en 1 temps.

### Fractures sur prothèse

Atteignent essentiellement le fémur, rarement l'acétabulum. Elles sont liées soit à un excès de force exercées sur l'os, soit à une diminunition de la résistance de celui-ci. La fréquence de ces fractures est diversement rapportée et semblent voisine de 1 à 2% [21], alors que dans notre série nous avons noté 3,2% de fracture de fémur sur PTH et aucun cas de fracture de cotyle. Le délai entre l'implantation et la survenue de la fracture est mal connu, notamment pour la tige non cimentée. Il a été évalué à 8 ans pour les tiges cimentées [22].

La prise en charge de ce type de fractures est difficile en raison de l'âge souvent avancé et de la fragilité des patients, de l'ostéoporose, et de la menace que ces fractures font peser sur la fixation de la prothèse parfois déjà défaillante. Dans notre série nous avons traitée 11 cas de fracture fémorale sur prothèse, par la reprise de la prothèse avec changement de la tige et mise en place d'une tige longue, ce traitement était utilisé pour les fractures stade C selon la classification de Vancouver, par plaque vissée avec cerclage ou cerclage simple pour les stades A et B.

### Luxations tardives

Définie par leur survenue d'une luxation de PTH au-delà de 5ans [23]. La luxation est, après le descellement, la 2éme complication susceptible de remettre en cause le résultat d'une arthroplastie de la hanche, sa fréquence selon les séries publiées se situe entre 0,11% et 9% [24] (Tableau 6). Plusieurs mécanismes, parfois associés, peuvent être invoqués: faiblesse musculaire qui augmente avec l'âge; la distension de la capsule chez les patients ayant récupéré une grande mobilité et sa fragilisation par la réaction macrophagique réactionnelle aux particules d'usure [27] un épanchement intra-articulaire réactionnel à ces particules d'usure; une déformation de bord de la cupule par des subluxations répétées [28]; l'usure de la cupule. Les résultats de la littérature ont déterminés plusieurs facteurs favorisants:

**Tableau 6:** La fréquence de luxation de prothèse totale de la hanche dans les différentes séries

Auteurs	série	Nombre de cas de luxation	Fréquence %
Ray A [2]	1000	17	1,7%
Masaoka T [25]	317	10	3,2%
Woo RY [26]	10500	325	3,2%
Notre série	215	11	5,11%

### Terrain

**Age**: plusieurs séries ont permis d'apprécier la fréquence de l'instabilité prothétique chez les patients de plus de 80 ans, ainsi le grand d'âge est un facteur reconnu d'instabilité prothétique [29], sans doute explicable par la diminution de la force musculaire, les pathologies associées et le non-respect des précautions d'usage. Le risque de luxation est ainsi dix fois plus élevé chez les patients ayant un score ASA (American society of anesthesia) supérieur ou égal à 3 [30].

**Sexe**: pour WOO et Morrey [26] le taux de luxation est significativement plus élevé chez la femme (3,8% contre 2,5%). Cette différence pour être attribuée à une plus grande faiblesse musculaire et une plus grande mobilité.

**Pathologies neuromusculaires**: nombreux auteurs ont mis en évidence le rôle néfaste des syndromes neurologiques (hémiplégie, maladie de Parkinson, épilepsie) et des troubles psychiques (démence sénile, encéphalopathie éthylique) [31, 32].

### Traitement des luxations tardives

**Réduction**: Dans notre série nous avons traitée 7 cas de luxation tardive, la réduction était réalisée sous AG dans 3 cas compléter par une traction pendant 6 semaines puis la rééducation. Nous avons noté la récidive de luxation dans 1 cas. La revue de la littérature montre que le risque de récidive après traitement orthopédique d'une première luxation est diversement apprécié: 17% pour Fraser et Al [33]. 25% pour Turner [34] et 34% pour Garcia-Cimberto [35]. La récidive est d'autant plus fréquente que la luxation a été plus tardive [36] Qu'elle a été réduite orthopédiquement et qu'il existe des facteurs d'instabilité reconnus: arthroplastie itérative, pseudarthrose trochantérienne, malposition.

**Le traitement chirurgical**: Le traitement était chirurgical dans 4 cas dans notre série.

**Changement de position de cotyle dans 3 cas:** dans 2 cas le cotyle était cimenté dont son ablation était difficile mais sans dégâts osseux. Nous avons réalisé le changement de position et de l'orientation de cotyle dans les 3 cas avec réimplantation au niveau de son ancienne cavité sans anneau de reconstruction ni greffe osseuse. Nous avons noté une bonne évolution des patients traités. Nous avons réalisé dans un cas une reconstruction de cotyle par anneau de Kq1erboull chez un patient traité par PTH pour dysplasie du cotyle. Le traitement chirurgical doit être envisagé rapidement s'il existe une cause de luxation dont le traitement ne peut être que chirurgical (malposition importante, déplacement de grand trochanter') et, dans le cas contraire, à partir de la deuxième ou la troisième luxation [36] d'autant plus qu'elle (s) survient (surviennent) rapidement après la première.

**Remplacement prothétique**: un défaut de position important de la cupule impose son changement, qui permet de réorienter le cône de la mobilité de la prothèse. Il serait en cause dans 1/3 des cas pour Garcia et al [37]. L'ablation de la cupule peut être difficile et ne doit pas être créé des lésions osseuses. La réimplantation de la nouvelle cupule peut faire appel aux techniques de réimplantation utilisées dans les descellements aseptiques (renforcement métallique, associé souvent à une reconstruction par greffe osseuse). La nouvelle cupule sera implantée en corrigeant la malposition, ce qui suppose de l'avoir soigneusement identifiée et d'avoir pris des repères très précis permettant de positionner correctement la nouvelle cupule. Le résultat de la littérature [38] a montré que les malpositions fémorales sont moins fréquentes et le changement de cette pièce pose plus de problème technique. L'extraction d'une tige non descellée peut être très difficile et créer des lésions osseuses.

## Conclusion

Les complications tardives sont très graves. Elles peuvent avoir aussi un retentissement fonctionnel grave sur l'état physique et psychique de patient. Leur prise charge est souvent difficile et compliquée, et nécessite une chirurgie prothétique sophistiquée, un chirurgien compétent et une coopération de patient pour avoir des résultats satisfaisants.
